# SWATH-MS based quantitive proteomics reveal regulatory metabolism and networks of androdioecy breeding system in *Osmanthus fragrans*

**DOI:** 10.1186/s12870-021-03243-8

**Published:** 2021-10-13

**Authors:** Yi-Fan Duan, Cheng Zhang, Min Zhang, Yu Ye, Kai-Lu Zhang, Mo-Xian Chen, Lin Chen, Xian-Rong Wang, Fu-Yuan Zhu

**Affiliations:** 1 College of Biology and the Environment, International Cultivar Registration Center for Osmanthus, Nanjing Forestry University, Nanjing, 210037 China; 2 College of Biology and the Environment, Co-Innovation Center for Sustainable Forestry in Southern China, Nanjing Forestry University, Nanjing, 210037 China

**Keywords:** androdioecy, SWATH-MS, Proteomics, Pistil, *Osmanthus fragrans*

## Abstract

**Background:**

The fragrant flower plant *Osmanthus fragrans* has an extremely rare androdioecious breeding system displaying the occurrence of males and hermaphrodites in a single population, which occupies a crucial intermediate stage in the evolutionary transition between hermaphroditism and dioecy. However, the molecular mechanism of androdioecy plant is very limited and still largely unknown.

**Results:**

Here, we used SWATH-MS-based quantitative approach to study the proteome changes between male and hermaphroditic *O. fragrans* pistils. A total of 428 proteins of diverse functions were determined to show significant abundance changes including 210 up-regulated and 218 down-regulated proteins in male compared to hermaphroditic pistils. Functional categorization revealed that the differentially expressed proteins (DEPs) primarily distributed in the carbohydrate metabolism, secondary metabolism as well as signaling cascades. Further experimental analysis showed the substantial carbohydrates accumulation associated with promoted net photosynthetic rate and water use efficiency were observed in purplish red pedicel of hermaphroditic flower compared with green pedicel of male flower, implicating glucose metabolism serves as nutritional modulator for the differentiation of male and hermaphroditic flower. Meanwhile, the entire upregulation of secondary metabolism including flavonoids, isoprenoids and lignins seem to protect and maintain the male function in male flowers, well explaining important feature of androdioecy that aborted pistil of a male flower still has a male function. Furthermore, nine selected DEPs were validated via gene expression analysis, suggesting an extra layer of post-transcriptional regulation occurs during *O. fragrans* floral development.

**Conclusion:**

Taken together, our findings represent the first SWATH-MS-based proteomic report in androdioecy plant *O. fragrans*, which reveal carbohydrate metabolism, secondary metabolism and post-transcriptional regulation contributing to the androdioecy breeding system and ultimately extend our understanding on genetic basis as well as the industrialization development of *O. fragrans*.

**Supplementary Information:**

The online version contains supplementary material available at 10.1186/s12870-021-03243-8.

## Background

Androdioecy is an exceedingly rare mating system in which males and hermaphrodites co-occur in a single population with normal sexual function [1]. Androdioecious plants are extremely rare in nature, less than 50 plants have been reported worldwide such as *Datisca glomerata* [2], *Mercurialis annua* [3], *Pseudoxandra spiritus-sancti* [4] and *Tapiscia sinensis* [5, 6], drawing widespread interests by scientists. Surprisingly, such an unusual breeding system prefers to be distributed in the Oleaceae species including *Phillyrea latifolia* [7], *Phillyrea angustifolia* [8], *Fraxinus lanuginose* [9], *Chionanthus retusus* [10], *Osmanthus serrulatus* [11], *O. delavayi* [12] etc., accompanied by higher frequencies of males compared to other androdioecious species [13]. *Osmanthus fragrans* Lour. belongs to *Osmanthus* of Oleaceae, with important dietary value and economic value, widely used in food processing industry through their fragrant flowers. *O. fragrans* has two sex traits, the male plant has an abortive pistil and a normally developed stamen, the other is the hermaphroditic plant with normal development of both stamen and pistil, which is a proper model to investigate androdioecy [14]. Nevertheless, the molecular mechanism of reproductive development and sex differentiation in androdioecy plants is still largely unknown and requires more detailed investigations.

Since sex differentiation has recently become a research hotspot, proteomics has been demonstrated as a powerful tool to examine sex-related differences in plants and even the stress adaption changes from dioecious plants [15]. For example, comparative proteomic analysis of the mitochondrial proteome between sterile rice and fertile rice revealed a clade of regulatory proteins with decreased abundance in mitochondrial complex leading to cytoplasmic male sterility in sterile rice [16]. A gel-based proteomics study on male and female leaves of jojoba (*Simmondsia chinensis*) established 45 molecular protein markers for early sex differentiation [17]. Similarity, proteomics analysis of male and female pumpkin nectar identified a total of 12 specific proteins unique to female and male nectar [18]. The total proteome profiling of flower buds of different genders was analyzed using label-free proteomics methods, providing a basis for identifying the key proteins in the development of male and female flowers of *Coccinia grandis* [19]. Interestingly, proteome changes of male and female *Populus cathayana* under drought stress were investigated by two-dimensional electrophoresis (2-DE) method, the upregulation of photosynthesis, energy metabolism and stress responses in male poplars explaining the observation that male poplars are more resistant to drought stress compared to female poplars [20]. Such phenomenon also occurred in other abiotic stresses such as salt, chilling and heavy metals [21–24]. Moreover, large-scale proteomic analysis has been widely used in many other plant research as a promising tool for protein identification and gene function characterization [25, 26].

The innovative SWATH-MS (Sequential Windowed Acquisition of All Theoretical Mass Spectra) approach employs a high specificity data-independent acquisition (DIA) method coupled with a novel targeted data extraction methodology, displays the advantages of higher reproducibility, quantitative consistency and accuracy compared with gel-based and label-based or defined DDA proteomic approaches [27–29], particularly for detecting and quantifying low abundant proteins, which has always been the major challenges in the proteomics investigation [30, 31]. In this study, a total of 2298 proteins were quantitatively identified by SWATH-MS in *O. fragrans* flowers, 428 of which were found to be differentially expressed between male and hermaphroditic pistils. Alternations were observed in different metabolic processes such as glucose metabolism, flavonoids and isoprenoids metabolism, related signaling pathway, which contributes to the normal developmental growth of anthers and pollen tube. Further experimental analysis revealed that hermaphroditic *O. fragrans* could accumulate more carbohydrates than males during the full flowering stage. Our work represents the first SWATH-MS investigation in woody plant *O. fragrans*, hoping to provide a comprehensive proteome reference for the unique androdioecy breeding system from the perspective of proteomics.

## Results

### Phenotype characterization for appropriate sampling and following SWATH-MS analysis

**Fig. 1:**
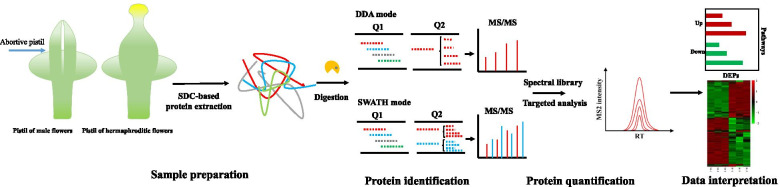
Simplified systematic workflow for the SWATH-MS-based proteomic analysis of male and hermaphroditic flowers

To perform a comprehensive proteomes analyses on androdioecy breeding system in *Osmanthus fragrans*, we used innovative SWATH-MS-based quantitative proteomic strategy to compare the proteomes of male and hermaphroditic flowers as outlined in Fig. 1, which employs a high specificity data-independent acquisition (DIA) method coupled with a novel targeted data extraction methology. The acquired DIA file recording all the complete chromatographic elution traces of peak groups was introduced into a non target analysis by the MS2-based quantification methods, which provides continuous and sufficient information in protein quantification. Hence, the quantitative analyses of peptides were supported by given extracted ion chromatograms from both MS1 and MS2 level, enabling comprehensive proteome profiling and detecting low-abundance proteins in this study.

**Fig. 2:**
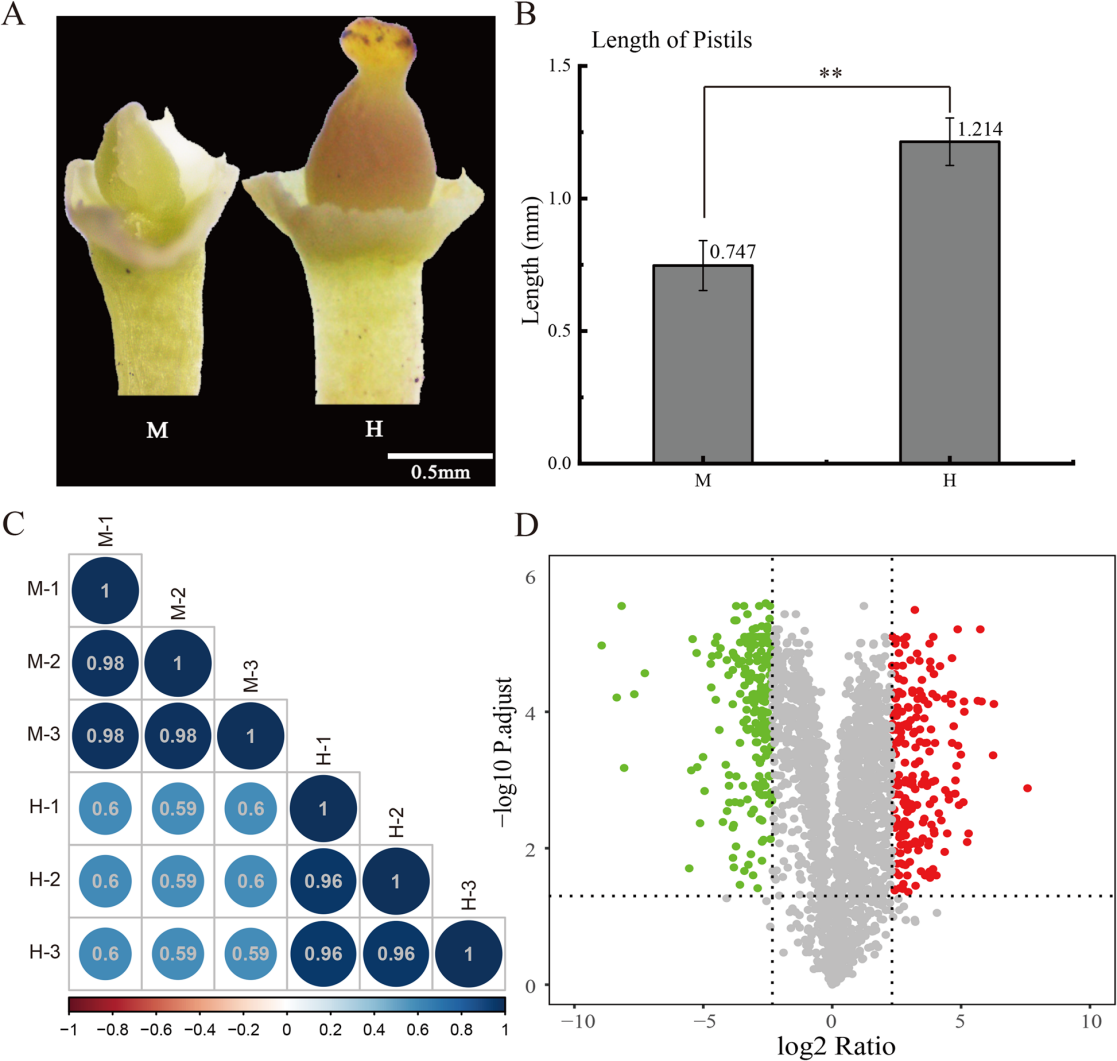
The appropriate sampling in this study and an overview of quantitative proteomic data. **a**. Phenotypes of male and hermaphroditic flower pistils (M, H). **b**. Pistils length of M and H, the pistil length was measured with electronic vernier caliper, and each was repeated 6 times. Data are presented as mean ± SD. **Significantly different from M data (*p* < 0.01 by Student’s t test). **c**. Correlation analysis of M and H quantitative proteomic data. **d**. The volcano map of the DEPs, the green dots represented down-regulated proteins and red dots indicated up-regulated proteins

Both male and hermaphroditic flowers of *O. fragrans* were sampled at the full bloom stage, and other floral organs were removed manually, leaving only the pistils. The hermaphroditic flower pistil has three complete organs: papillary stigma, shorter style, and enlarged ovary. Nevertheless, the pistil of male flowers was aborted, two carpels were separated without these three parts, accounting for pistil abortion. Simultaneously, the pistils of male flowers were significantly shorter than that of hermaphroditic flowers, 0.75 mm and 1.21 mm, respectively (Fig. 2a, b). Therefore, the pistils of male and hermaphroditic flowers were used in this proteome study by the SWATH-MS method. The correlation coefficient of the samples within the group (M and H) was generally higher than 0.95 whereas the correlation between the groups was relatively lower at around 0.6 (Fig. 2c), strongly reflected the high reliability of the appropriate sampling and suitable for subsequent analysis. In this study, a total of 2298 proteins were identified in male and hermaphroditic flower pistils (Table S1), among which 428 proteins showing significant changes in protein abundance including 210 increased and 218 decreased in the male compared to hermaphroditic pistils as shown in the volcano map (Fig. 2d).

### Comprehensive inventory of proteome changes between male and hermaphroditic flowers

**Fig. 3:**
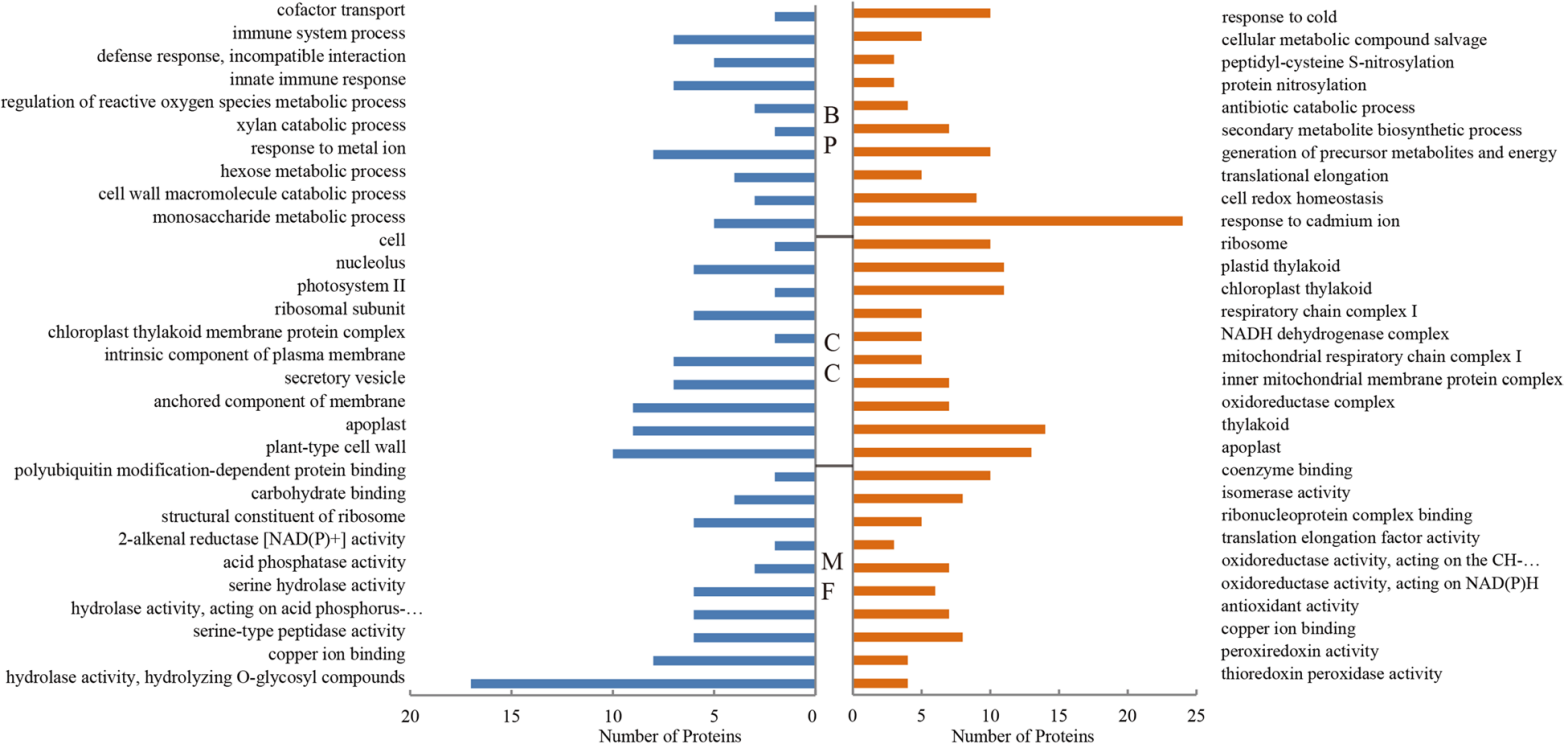
Gene Ontology (GO) analysis of the DEPs. Blue and orange respectively represent the top 10 BP, CC and MF of down-regulated and up-regulated proteins in order by p-value

The functional classifications of these DEPs were carried out through Gene Ontology (GO) analysis including biological processes (BP), cellular components (CC) and molecular functions (MF). The top terms corresponding to the up-regulated DEPs of BP/CC/MF were response to cadmium ion, thylakoid, and coenzyme binding whereas the top terms corresponding to the down-regulated DEPs of BP/CC/MF were response to metal ion, plant-type cell wall and hydrolase activity, hydrolyzing O-glycosyl compounds (Fig. 3). In BP terms for up-regulated and down-regulated proteins, “response to cadmium ion” and “response to metal ion” constitute the largest number of proteins in both cases, as revealed by previous studies that *O. fragrans* has resistance to metal ions such as cadmium, lead and copper [32, 33]. However, there were few studies on the molecular response and resistance mechanisms of *O. fragrans* to heavy metals still requiring further investigations. It is also worth noting that massive down-regulated proteins are assigned with the “hydrolase activity” and “hydrolyzing O-glycosyl compounds” MF term in the male flower, implicating their potential roles for normal pistils development.

**Fig. 4:**
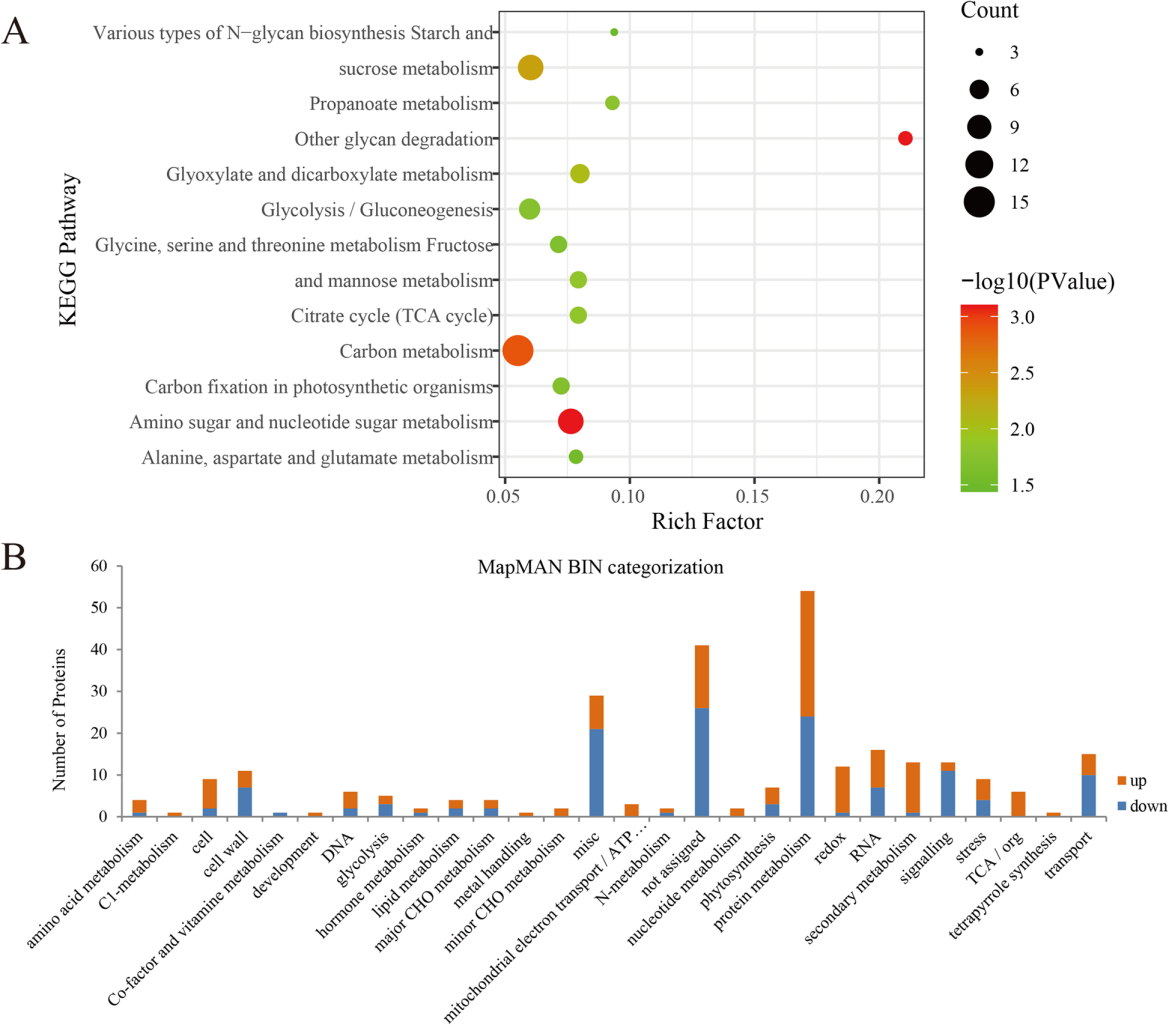
KEGG and MapMAN BIN categorization of DEPs. **a**. KEGG pathway enrichment of the DEPs. **b**. MapMAN BIN categorization of DEPs, blue and orange represent down-regulated and up-regulated proteins, respectively

All the DEPs were mapped and enriched in top 13 pathways such as carbon metabolism, amino sugar metabolism and starch metabolism by KEGG pathway classification (Fig. 4a), which provide a quick view of the most significant pathway in the female sterility. Meanwhile, MapMAN BIN analysis was also conducted on all the DEPs (Table S2), revealing several metabolic processes including protein metabolism, RNA processing, transport, and secondary metabolism were greatly influenced during the pistil abortion of male *O. fragrans* flowers (Fig. 4b). In particularly, 12 of the 13 DEPs involving secondary metabolism were up-regulated while most of signaling related proteins were down-regulated proteins in the male pistils. Such completely opposite protein expression patterns in these two categories between male and hermaphroditic pistils, which may be the key response attribute to the pistil abortion of male flowers. In the following sections, those activated or repressed enzymes will be mapped to different physiological and biochemical pathways, and the relevance of diverse biological processes and molecular androdioecy characteristics of *O. fragrans* is discussed.

### Protein-protein interaction (PPI) network analysis of DEPs

**Fig. 5:**
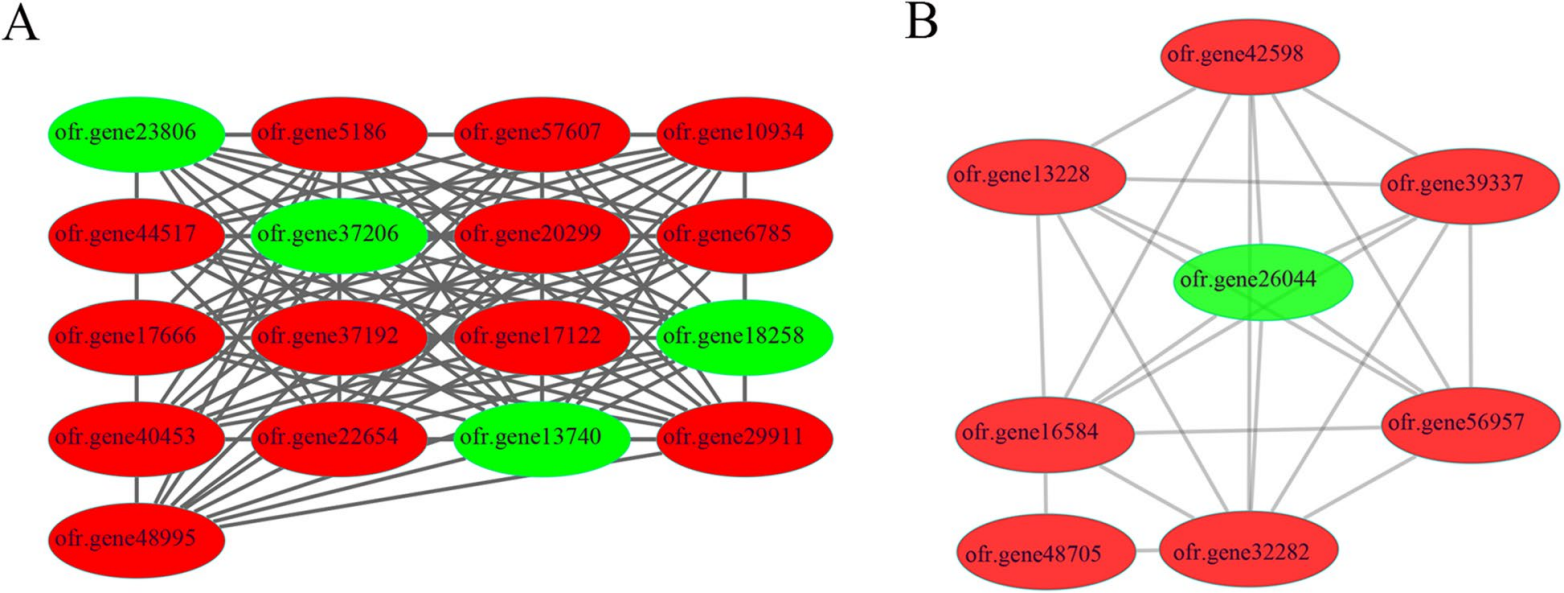
Protein-protein interaction (PPI) network analysis of DEPs. **A** and **B** were the top two PPI network modules in MCODE analysis. The red and green nodes represented up- and down-regulated proteins, respectively

The study of protein-protein interaction (PPI) is beneficial to explore the core proteins and better understand the protein regulatory network. We used 428 DEPs for protein-protein interaction network analysis, and the two main protein interaction modules were screened by MCODE (Fig. 5). In the first module, there were 17 nodes and 117 edges, of which the key protein was ofr.gene22654, an enrichment factor protein (Fig. 5a). Its knockout mutant can significantly reduce seed setting rate in Arabidopsis, only about 40% of the wild type [34]. In the second module, there were a total of 8 nodes and 23 edges. Among all 8 proteins, the protein ofr.gene26044 is the only down-regulated protein that plays a role in the synthesis of Jasmonic acid (Fig. 5b). As a signaling molecule, jasmonic acid is deeply involved in the growth and development of plants. Many studies have shown that the decrease of JA content caused by JA synthesis related gene mutation can lead to female infertility in tomato (*Solanum lycopersicum*) [35–37].

### Correlation analysis between differentially expressed gene and protein in male and hermaphroditic pistils

**Fig. 6:**
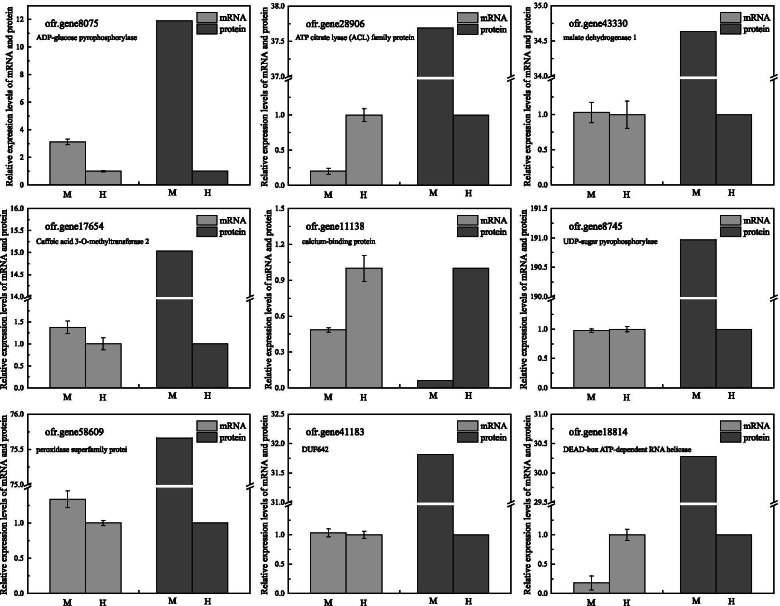
Correlation analysis between differentially expressed gene and protein in male and hermaphroditic pistils. Nine proteins identified by SWATH-MS were selected to examine the transcript level by qRT-PCR analysis in male and hermaphroditic pistils

To understand whether the expression of differentially expressed proteins are associated with transcriptional changes, we selected 9 genes involving a variety of processes for expression levels analysis by qRT-PCR. As shown in Fig. 6, four of the selected genes showed up- or down- regulation in accordance with the changes in abundance of the corresponding proteins identified from SWATH-MS experiment (Table S1), which are distributed in carbohydrate metabolism, secondary metabolism, signaling pathway as well as cell wall synthesis. However, two genes including ofr.gene28906 and ofr.gene18814 were expressed at the opposite level of protein expression (Fig. 6). Such difference between transcription levels and protein expression levels may be resulting from post-transcriptional regulation such as alternative splicing (AS), mRNA stability, and mRNA translation [38]. Additionally, ofr.gene43330, ofr.gene8745 and ofr.gene41183 were all up-regulated in the proteomic data, but their gene expression was slightly up-regulated or no significant difference in the male compared to hermaphroditic pistil (Fig. 6), implicating a complex and delicate regulation stage from the transcriptome to the proteome.

## Discussion

### Carbohydrate metabolism and TCA cycle in pistils

Fertilization of higher plants depend on the pollen tube elongation of the style toward the ovary, which was also considered to be the fastest growing plant cell requiring a huge consumption of nutrient and energy [39, 40]. Since the DEPs primarily focused on the carbohydrates and amino acids metabolism pathways, such gap probably restricted the normal developmental growth of pollen tube in male flowers, resulting in the disruption of pollen germination at stigma and complete fertilization. Consistently, down-regulation of major carbon and sugar metabolism responsible for the nutrient and energy supply were observed in male plants with incomplete pistil (Table S2).

**Fig. 7:**
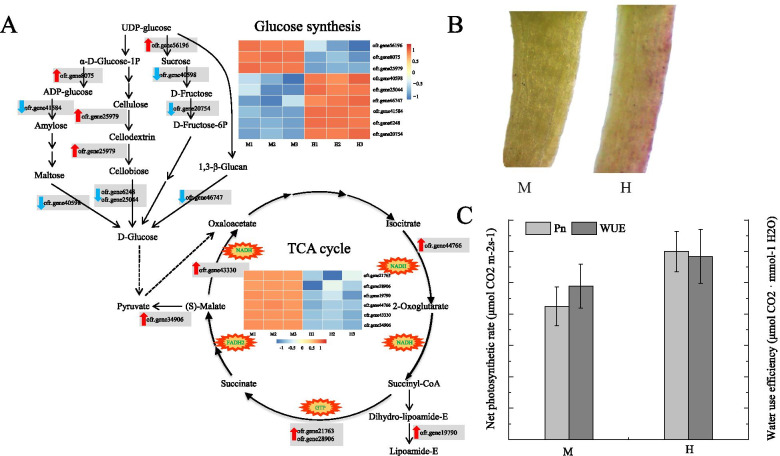
Carbohydrate metabolism and TCA cycle in pistils. **a**. Starch and sucrose metabolism pathway and TCA cycle of DEPs. The red upward arrows and the blue downward arrows indicated the up-regulated and down-regulated proteins involved in these two pathways, respectively, and their relative expressions were displayed in the heat maps. **b**. Morphology of male and hermaphroditic flower pedicel (M, H). **c**. Net photosynthetic rate (Pn) and water use efficiency (WUE) of male and hermaphroditic flowers at the full flowering stage

In the starch and sucrose metabolism, UDP-glucose eventually converted to D-glucose, 4 up-regulated proteins and 7 down-regulated proteins involving this pathway were identified in male flower compared with hermaphroditic flower pistils (Fig. 7a). The up-regulated proteins promoted the conversion of UDP-glucose into other forms of carbohydrate and the down-regulated proteins inhibited the synthesis of D-glucose. Therefore, the UDP-glucose and glucose in male flower were both reduced in comparison to hermaphroditic flowers. As UDP-glucose functions as metabolic precursors of cellulose for the inner layer of pollen tube cell wall formation [41], the reduced contents of glucose may be the main cause for the abortive pistil in male *O. fragrans* flowers without complete pistil structure such as stigma, style and ovary.

The TCA cycle is a crucial pathway connection for sugar, lipid, and protein metabolism. KEGG pathway analysis and MapMAN BIN classification simultaneously revealed significant changes in the TCA cycle with 6 identical up-regulated proteins in male pistils (Fig. 4b, Fig. 7a), which well mapped to each reaction step involving the TCA cycle within the generation of different energy related products. In fact, the promoted TCA cycle accelerated the consumption of glucose, further restricting the development of pollen tube cell walls, thus affecting pistil growth in male flower through the deficiency of nutrient and energy supply.

Moreover, the full bloom stage is the main stage of flowering and pollination. In this process, the pedicel of hermaphroditic flower gradually turns purplish red whereas the male remains green (Fig. 7b), strongly implicating the accumulation of carbohydrate-related compounds in hermaphroditic flower. To further detect the carbohydrate metabolism distribution, LI-6400XT portable photosynthetic measurement system was performed to determine photosynthesis of male and hermaphroditic *O. fragrans* during the full flowering stage (Table S4). The results showed that the net photosynthetic rate and water use efficiency in hermaphroditic flower were higher than that of males (Fig. 7c), indicating that hermaphroditic *O. fragrans* could accumulate more carbohydrates with less water consumption to fulfill pollen germination, pollen tube growth and following fruit development in accordance with the results of the above proteome data.

### Up-regulation of secondary metabolism for promoting male function in male flowers

**Fig. 8:**
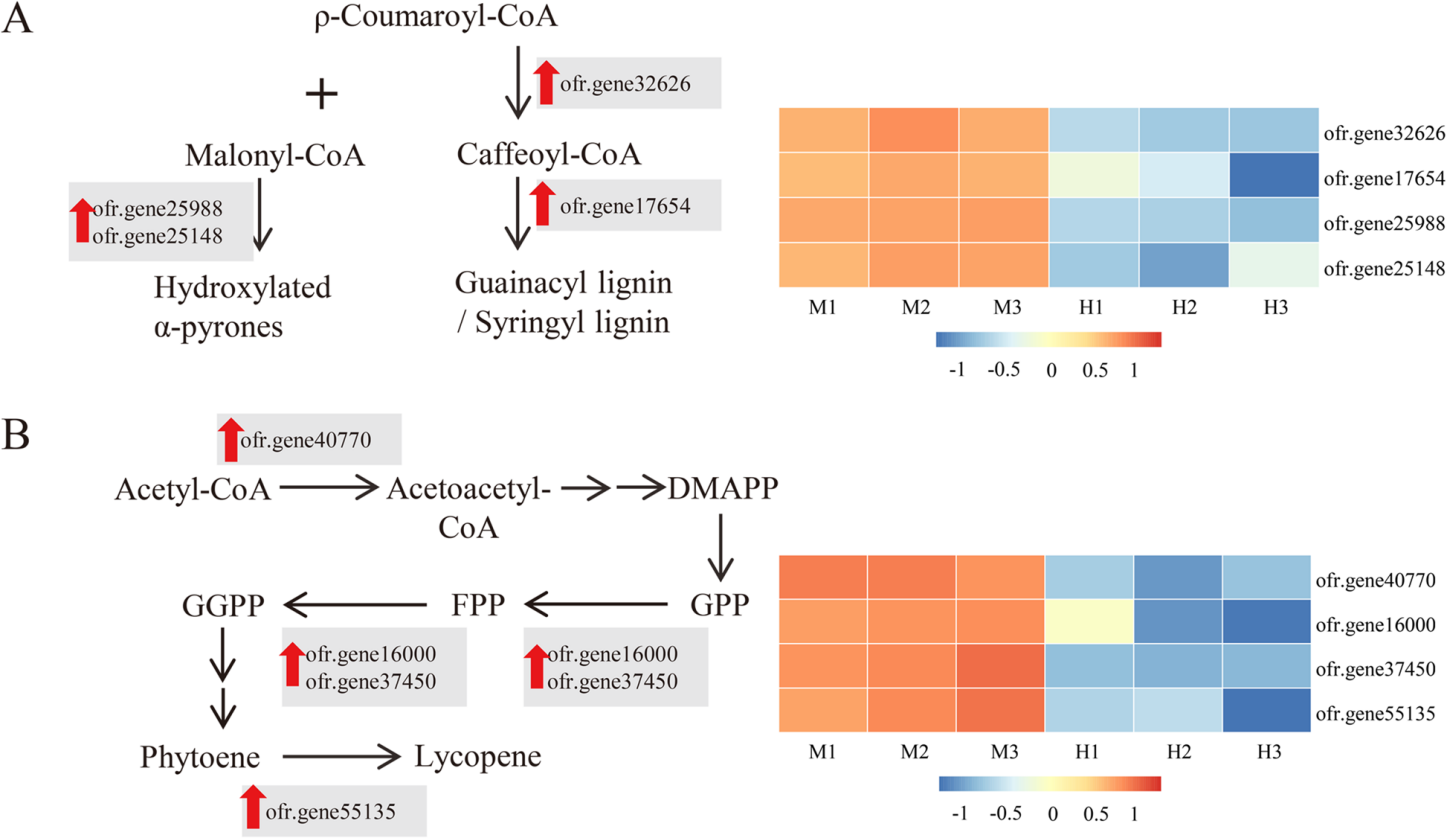
Up-regulation of secondary metabolism in male flowers. **a**. Up-regulated proteins involving the metabolic pathways of lignin and flavonoids. **b**. Up-regulated proteins in isoprenoids metabolic pathways

By MapMAN BIN and KEGG categorizations, a large proportion of proteins involving the secondary metabolism was shown to be activated in male flowers, particularly for the biosynthesis of secondary metabolites such as flavonoids, isoprenoids and phenylpropanoids, etc. (Fig. 8). Generally, the precursors of flavonoids and isoprenoids were both p-coumaroyl CoA in the phenylpropanoids pathway, which was synthesized by chalcone synthase (CHS) and hydroxycinnamoyl-CoA shikimate/quinate hydroxycinnamoyl transferase (HCT), respectively [42]. The ofr.gene25988 and ofr.gene25148 are the key enzymes for the synthesis of flavonoids and their homologs namely as less adhesive pollen 5 (LAP5) and LAP6, respectively, were essential for the formation of the pollen exine in Arabidopsis [43]. Meanwhile, the acetoacetyl-CoA thiolase (AACT) ofr.gene40770 catalyzes the conversion of acetyl-CoA to acetoacetyl-CoA for isoprenoids metabolic pathway. Mutation of its homolog in Arabidopsis could lose the pollen coat and tapetal cells leading to male sterility [44]. Accordingly, the flavonoids and other substances are important components of the anther or pollen tapetum to ensure the fertility of males, playing an important role in the attachment and recognition of pollination [45]. The absence of flavonoids severely influence pollen germination and pollen tube growth without any damage to the size and structure of pollen resulting in male sterility [46]. DEPs participating in the flavonoids and isoprenoids biosynthesis pathway were up-regulated by 9.3-fold on average in the pistil of male flowers for ensuring the fertility of pollen (Fig. 8), feasible to explain important features of androdioecy that even the pistil of the male flower has been aborted, whose pollen still remains vigorous and can pollinate with the fertile pistil of other hermaphroditic flowers. And previous studies believe that the fitness of male function in the male plant was at least twice as high as that of the hermaphroditic plant in the androdioecy breeding system in order to maintain its existence in nature [47, 48]. Therefore, those important secondary metabolites including flavonoids, isoprenoids and lignin are likely to be protective a role for maintaining male function in male flowers.

### Down-regulation of signaling related proteins in pistils of male flowers

In contrast to secondary metabolism, DEPs in the signaling categorization showed reduced protein abundances changes, most of which were highly correlated with calcium signaling, receptor-like kinase (RLK) and leucine rich repeat receptor-like kinase (LRR-RLK) (Table 1; Table S2). It was well known that calcium is an important second messenger involving various signal transduction pathways, particular for stress response and reproductive growth in plants [49]. Free calcium is essential for pollen germination, pollen tube growth, and pollen tube guidance [50]. The dynamic changes of calcium content were investigated during the pistil development of olive (*Olea europaea* L.), revealing calcium gradually accumulated on the stigma and further expanded toward the style and ovary after the anthers dehiscence, implicating the growth direction of the pollen tube was determined by the concentration of calcium [51]. Meanwhile, distribution changes of calcium regulate programmed cell death to affect the differentiation of male and female flowers in litchi (*Litchi chinensis* Sonn.) such as the degeneration of style cells by the absence of calcium precipitation [52]. Therefore, down regulation of calcium signaling (ofr.gene26367, ofr.gene11138 and ofr.gene18811) probably attributed to the degeneration of pistils in male flowers. On the other hand, the CrRLK gene family has been widely demonstrated to be associated with the tip growth and cell wall integrity of pollen tubes during fertilization [53, 54]. For instance, the Arabidopsis knockout mutant feronia (FRE) belonging to CrRLK family failed to release sperm cells and impair pollen tube reception [55]. The Buddha’s Paper Seal 1 (BUPS1) and BUPS2 were also required for the pollen tube development and disruption of them caused the swollen of pollen tube unable to complete fertilization [56]. Recently, 26 CrRLK genes in the pear genome were identified, among which PbrCrRLK1L26 regulate pollen tube elongation and PbrCrRLK1L3 could control pollen tube rupture [57]. Accordingly, we identified two CrRLK (ofr.gene38451 and ofr.gene2855) may modulate pollen tubes development responsible for the aborted pistils of male flowers in *O. fragrans*. Taken together, entire down-regulation of both calcium signaling and RLK related proteins probably severely restrict the pistils morphogenesis including pollen tube elongation, sperm cells guidance after rupture as well as carpel fusion, leading to female sterility in male flowers.

**Table 1:** DEPs related to signaling in MapMAN BIN system

Protein	MapManBin	Bin Name	Ratio (M/H)
ofr.gene26367	30.3	calcium	0.16
ofr.gene11138	30.3	calcium	0.06
ofr.gene18811	30.3	calcium	0.15
ofr.gene49445	30.8	misc	0.15
ofr.gene38451	30.2.16	receptor kinases. Catharanthus roseus-like RLK1	0.18
ofr.gene2855	30.2.16	receptor kinases. Catharanthus roseus-like RLK1	0.17
ofr.gene58949	30.2.3	receptor kinases.leucine rich repeat III	0.06
ofr.gene15871	30.2.3	receptor kinases.leucine rich repeat III	0.18
ofr.gene46838	30.2.3	receptor kinases.leucine rich repeat III	0.15
ofr.gene12356	30.2.7	receptor kinases.leucine rich repeat VII	0.15
ofr.gene14924	30.2.14	receptor kinases.leucine rich repeat XIV	0.14

### Other candidate proteins contribute to androdioecy breeding system in *O. fragrans*

Seeking target proteins for the differentiation of male and female flowers in *O. fragrans* is necessary to illuminate the molecular mechanism of androdioecy breeding system. Here, we collected 25 candidate proteins with dramatic abundance changes (Ratio > 30 or Ratio < 0.03) between M and H as shown in Table 2. Representatively, UDP-sugar pyrophosphorylase (ofr.gene8745, USP) was the largest up to 190 folds of up-regulation in the male flowers, which has been reported to be essential for the recycling of xylose and arabinose. Mutation of USP exhibited the incomplete cell wall of anther and pollen leading to sterility in Arabidopsis [58]. Furthermore, ofr.gene58609 and ofr.gene10710 participates in the biosynthesis of lignin, which is critical for cell wall remodeling and modification in pollen development [59, 60]. Therefore, these proteins showing very high abundance in the male pistil are probably guaranteed to maintain male fertility, and improve the competitiveness and compatibility of male pollen. On the other hand, the lowest expression of candidate protein (ofr.gene35198 and ofr.gene10041) in the pistils of male flower compared to the hermaphroditic flower encodes pectin lyase-like and pectin acetylesterase activity, emerging evidences have demonstrated that pectin is the main component of the apical cell wall, which contributes to the mechanical strength of the cell wall and plays an important role in the growth of pollen tubes which may be associated with the pistil abortion of *O. fragrans* [61, 62].

**Table 2:** List of candidate DEPs with Ratio > 30 or Ratio < 0.03

Protein	TAIR ID	Description	Ratio (M/H)
ofr.gene8745	AT5G52560	UDP-sugar pyrophosphorylase	190.972
ofr.gene55222	AT5G13450	delta subunit of Mt ATP synthase	77.311
ofr.gene58609	AT5G05340	Peroxidase superfamily protein	75.664
ofr.gene29794	AT1G24020	MLP-like protein 423	55.531
ofr.gene19061	AT4G29270	Acid phosphatase-like protein	53.622
ofr.gene44766	AT4G35650	isocitrate dehydrogenase III	50.243
ofr.gene33663	AT1G70830	MLP-like protein 28	39.128
ofr.gene28906	AT2G20420	ATP citrate lyase (ACL) family protein	37.688
ofr.gene20390	AT5G63190	MA3 domain-containing protein	34.902
ofr.gene43330	AT1G53240	Lactate/malate dehydrogenase	34.634
ofr.gene10710	AT2G41380	S-adenosyl-L-methionine-dependent methyltransferases	34.003
ofr.gene41183	AT5G11420	Encodes a DUF642 cell wall protein	31.812
ofr.gene18814	AT3G53110	DEAD-box ATP-dependent RNA helicase 38	30.279
ofr.gene33622	AT1G17860	Kunitz trypsin inhibitor 5	0.029
ofr.gene10041	AT2G43870	Pectin lyase-like superfamily protein	0.027
ofr.gene19072	AT3G16240	Aquaporin TIP2–1	0.026
ofr.gene26948	AT2G21100	Dirigent protein 23	0.023
ofr.gene58659	AT5G07440	Glutamate dehydrogenase 2	0.023
ofr.gene6023	AT4G12080	AT-hook motif nuclear-localized protein 1	0.021
ofr.gene30313	AT4G39830	L-ascorbate oxidase	0.006
ofr.gene35198	AT4G19420	Pectin acetylesterase 8	0.005
ofr.gene47658	AT3G54040	PAR1 protein	0.004
ofr.gene51778	AT1G06620	1-aminocyclopropane-1-carboxylate oxidase homolog 1	0.003
ofr.gene37522	AT3G12700	Aspartic proteinase NANA, chloroplast	0.003
ofr.gene7688	AT5G55180	Glucan endo-1,3-beta-D-glucosidase	0.002

## Conclusion

In the androdioecy breeding system of *O. fragrans*, the pistils of male flowers were aborted without fully developed pistils but the pollen remains vigorous and can be pollinated whereas the pistil of the hermaphroditic flower developed normally. The present work provides a comprehensive understanding on the molecular androdioecy characteristics in *O. fragrans* by the advanced label-free SWATH-MS quantitative proteomics platform, which revealed significant proteome changes in carbohydrate metabolism, secondary metabolism as well as calcium signaling between M and H pistil. Accordingly, the reduced glucose metabolism cannot support normal pollen tube development leading to the aborted pistils in male flowers. On the contrary, sufficient carbohydrates accumulation in purplish red pedicel of hermaphroditic flower associated with experimental evidence of promoted net photosynthetic rate and water use efficiency in comparison to male flower (Fig. 7) further suggest glucose serves as nutritional modulator for the differentiation of male and hermaphroditic flower. Moreover, the entire upregulation of secondary metabolism including flavonoids, isoprenoids and lignin seem to protect and maintain the male function in male flowers, well explaining important feature of androdioecy that aborted pistil of a male flower still has a male function. In addition, down-regulation of calcium signaling and RLK related proteins also inhibit the pistil morphogenesis resulting in female sterility in male plants. Taken together, our work represents the first SWATH-MS-based proteomic study in androdioecy plant *O. fragrans*, which would provide new clues for further studies on the sex differentiation of the androdioecious *O. fragrans* and will extend our understanding on androdioecy breeding system.

## Methods

### Sample collection and preparation

Male and hermaphroditic flowers of *O. fragrans* were collected at their full flowering stage from the campus of Nanjing Forestry University. The development process of male and hermaphroditic *O. fragrans* is almost identical, and the main difference lies in the structure of pistil [14]. Thus, other tissues such as petals, pedicels, anthers were removed manually, only pistils of male and hermaphroditic flowers were left as experimental materials to eliminate the influence of other floral tissues as much as possible. The pistil length was measured with electronic vernier caliper, and each was repeated 6 times. The pistil samples of each sex for proteome detection had three repetitions, approximately 1 g per replicate. The treated pistils were fully ground in liquid nitrogen, added with 2.5% SDS/100 mm Tris-HCl lysis buffer. After 15 min of ultrasonic treatment on ice water, the supernatants were centrifuged at 16000 g for 20 min. Acetone was added to the supernatants to precipitate the protein. After cleaning with acetone and drying in the air, 8 M Urea / 100 mm Tris-HCl solutions were added to the protein precipitation to fully dissolve the proteins. After centrifuged at 12000 g for 15 min, the supernatants were added with dithiothreitol (DTT) to the final concentration of 10 mM, and incubated at 37 °C for 1 h. Iodoacetamide (IAM) was added until the final concentration of 40 mM, and the alkylation was carried out at room temperature in a dark state to seal the sulfhydryl group. Then, 100 mM Tris-HCl solutions were added, determining protein concentration by the Bradford method, and diluted urea concentration to less than 2 M. Trypsin (50:1 protein to trypsin) was added and oscillated at 37 °C overnight. The PH value of the solution was adjusted at about 6.0, then centrifuged at 12000 g for 15 min, and desalted with C18 columns. The desalted peptide solution was dried by a centrifugal concentrator and frozen at - 20 °C for mass spectrometry detection.

### SWATH-MS analysis

Peptides samples were detected by the Triple TOF 5600 (Sciex) LC/MS system. The prepared samples were bound to the trap column and then separated by the analytical column (45 min gradient, 60 min total time). Two mobile phases were established to analyze the gradient: Buffer A-0.1% (V/V) formic acid, 5% DMSO in H2O, Buffer B-0.1% (V/V) formic acid, and 5% DMSO in acetonitrile. For SWATH scanning, one MS1 scan (ion accumulation time 250 ms, scanning range 350–1500 m/z) and 100 MS2 scans with variable windows (ion accumulation time 33 ms, scanning range 100–1800 m/z) were included in each cycle. The mass spectrum files obtained by SWATH scanning were processed by DIA-Umpire to obtain the secondary mass spectrum file that can be used for database search. TPP software was used for database retrieval, and the retrieved results were used as a spectral library, and OpenSWATH algorithm was used for SWATH targeting extraction, and a false discovery rate (FDR) of <1% was set as selection criteria [63]. The protein quantitative intensity information obtained by SWATH analysis was applied to log2 conversion, data filling, and data normalization using the imputation algorithm in Perseus software for difference comparison and T-test analysis. Proteins of male and hermaphroditic pistils with a ratio of above 5 or below 0.2 (*P* < 0.05) were considered as differentially expressed proteins (DEPs) in this study.

### Proteomic data analysis

The correlation of protein quantification was analyzed by corrplot R package to evaluate the reliability of SWATH quantitative proteome data. The DEPs identified were used for Gene Ontology (GO) and Kyoto Encyclopedia of Genes and Genomes (KEGG) enrichment analysis by clusterProfiler R package [64], and pathways of KEGG enrichment analysis results is drawn with reference to KEGG mapper [65]. After the DEPs were compared with the homologous proteins in *Arabidopsis thaliana*, the MapMAN BIN system was used for functional classification (http://ppdb.tc.cornell.edu/dbsearch/searchacc.aspx). Heatmaps of DEPs between M and H were drawn by pheatmap R package [66]. Protein-protein interaction (PPI) network analysis of DEPs was carried out using String (https://string-db.org/), and the clustering function modules were screened by the Molecular Complex Detection (MCODE) plugin and visualized by Cytoscape [67, 68].

### Measurement of photosynthesis and quantitative real-time PCR analysis

The LI-6400XT portable photosynthesis measurement system was used to determine the photosynthesis of male and hermaphroditic *O. fragrans* at the full flowering stage according to the instructions. Four plants of each sex were selected, and 8 healthy and mature leaves of each plant were selected for measurement, and the net photosynthetic rate (Pn), water use efficiency (WUE) and other data were recorded.

Total RNA of male and hermaphroditic pistils were extracted with RNAprep Pure Plant Plus Kit (TIANGEN Biotech, Beijing) according to the manufacturer’s instructions. Then, 5 μg RNA was reversed transcribed by Evo M-MLV RT Premix for qPCR (Accurate Biotechnology, Hunan) for cDNA synthesis. SYBR Green Premix Pro Taq HS qPCR Kit (Accurate Biotechnology, Hunan) was used for quantitative real-time PCR (qRT-PCR) experiments with *OfACT* as a reference [69]. qRT-PCR was performed using ABI StepOnePlus Systems (Applied Biosystems, USA), the reaction steps were as follows: 95 °C for 30 s, followed by 40 cycles of 95 °C for 5 s and 60 °C for 30 s. All primers used in this experiment were listed in Table S3.

## Supplementary Information

**Additional file 1: Table S1.** Proteins identified by SWATH-MS in this study.

**Additional file 2: Table S2.** Functional classification of DEPs with the MapMAN BIN system.

**Additional file 3: Table S3.** Primers used for qRT-PCR experiment in this study.

**Additional file 4: Table S4.** Photosynthesis measurements of male and hermaphroditic O. fragrans at the full flowering stage.

## Data Availability

The data sets are included within the article and its additional files. The plant materials are available from the corresponding author on reasonable request.

## Abbreviations

SWATH-MS: Sequential Windowed Acquisition of All Theoretical Mass Spectra
DIA: Data-independent acquisition
DEPs: Differentially expressed proteins
GO: Gene Ontology
BP: Biological processes
CC: Cellular components
MF: Molecular functions
KEGG: Kyoto Encyclopedia of Genes and Genomes
TCA cycle: tricarboxylic acid cycle
CHS: Chalcone synthase
HCT: Hydroxycinnamoyl-CoA shikimate/quinate hydroxycinnamoyl transferase
LAP: Less adhesive pollen
AACT: Acetoacetyl-CoA thiolase
RLK: Receptor-like kinase
